# Stratified coastal ocean interactions with tropical cyclones

**DOI:** 10.1038/ncomms10887

**Published:** 2016-03-08

**Authors:** S. M. Glenn, T. N. Miles, G. N. Seroka, Y. Xu, R. K. Forney, F. Yu, H. Roarty, O. Schofield, J. Kohut

**Affiliations:** 1 Center for Ocean Observing Leadership, Department of Marine and Coastal Sciences, School of Environmental and Biological Sciences, Rutgers University, 71 Dudley Road, New Brunswick, New Jersey 08901, USA; 2 State Key Laboratory of Estuarine and Coastal Research, East China Normal University, 3663 Zhongshan Road North, Shanghai 200062, China; 3 Institute of Oceanology, Chinese Academy of Sciences, 7 Nanhai Road, Qingdao 266071, China

## Abstract

Hurricane-intensity forecast improvements currently lag the progress achieved for hurricane tracks. Integrated ocean observations and simulations during hurricane Irene (2011) reveal that the wind-forced two-layer circulation of the stratified coastal ocean, and resultant shear-induced mixing, led to significant and rapid ahead-of-eye-centre cooling (at least 6 °C and up to 11 °C) over a wide swath of the continental shelf. Atmospheric simulations establish this cooling as the missing contribution required to reproduce Irene's accelerated intensity reduction. Historical buoys from 1985 to 2015 show that ahead-of-eye-centre cooling occurred beneath all 11 tropical cyclones that traversed the Mid-Atlantic Bight continental shelf during stratified summer conditions. A Yellow Sea buoy similarly revealed significant and rapid ahead-of-eye-centre cooling during Typhoon Muifa (2011). These findings establish that including realistic coastal baroclinic processes in forecasts of storm intensity and impacts will be increasingly critical to mid-latitude population centres as sea levels rise and tropical cyclone maximum intensities migrate poleward.

## Abstract (web summary)

Accurate forecasts of hurricane intensity remain problematic. Here, using an ocean observing network to inform ocean and atmospheric model simulations, the authors show that consideration of ahead-of-eye cooling improves intensity forecasts.

## Graphical abstract

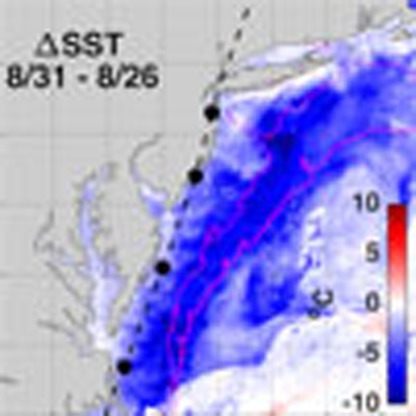

Tropical cyclones are among the most destructive weather phenomena on Earth^1^. Declines in hurricane related mortalities^2^ reflect improvements in global atmospheric and ensemble modelling approaches^3^ that have reduced hurricane track forecast errors by factors of 2–3 (ref. 4). Despite two decades of progress in hurricane track prediction, improvements in hurricane-intensity forecast skill have lagged significantly^4^. The predictions, public response and unexpected devastation patterns related to Hurricane Irene exemplify this dichotomy. Accurate track forecasts days in advance provided time for preparations and coastal evacuations, but Irene's official forecast maximum wind speeds along the Mid-Atlantic coast were consistently ∼5 m s^−1^ too high^5^. Irene instead caused catastrophic inland flooding because of heavy rainfall^5^, making it the eighth costliest cyclone to hit the United States since 1900 (ref. 6), with damages of ∼$16 billion (ref. 5). These intensity forecast uncertainties have significant negative consequences, ranging from unnecessary preparation costs to future public skepticism^7^.

Improved tropical cyclone intensity predictions include dependencies on the rapid space–time evolution of the atmosphere–ocean responses and feedbacks^8^. Coupled atmosphere–ocean models demonstrate that small shifts in sea surface temperature (SST) and stratification, even on small (100 km) horizontal scales, can have significant impacts on storm intensity^9^,^10^,^11^. Several studies have noted^12^,^13^,^14^,^15^,^16^ the relationship between warm and cold mesoscale features in the deep ocean and rapid changes in intensity, but the coastal ocean has received much less attention.

Here, utilizing an ocean observing network to inform ocean and atmospheric model simulations, the role of baroclinic processes on a stratified coastal ocean and their impact on the intensity of Hurricane Irene was quantified. The high percentage of ahead-of-eye-centre^14^,^17^,^18^ cooling (76–98%) observed in Irene is not reproduced by standard open ocean models that exclude these coastal baroclinic processes. Atmospheric model sensitivity studies indicate that intense in-storm sea surface cooling over a strongly stratified coastal regime is the missing contribution required to reproduce the rapid decay of Hurricane Irene's intensity. The 30-year historical buoy record shows an average of 73% of the in-storm cooling occurs ahead-of-eye-centre on the Mid-Atlantic Bight (MAB) in the stratified season. A Yellow Sea buoy observed up to 85% of in-storm cooling ahead-of-eye-centre during Super Typhoon Muifa (2011). The results demonstrate the importance of rapid ahead-of-eye-centre vertical shear-induced mixing processes and the ensuing ocean–atmosphere feedbacks for generating more accurate simulations of storm intensity.

## Results

### Synoptic conditions

**Figure 1:**
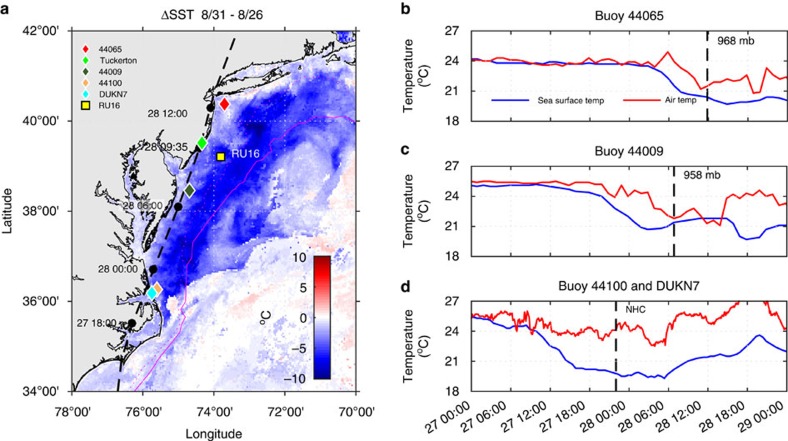
Map of the study domain with satellite and buoy data. (**a**) SST difference map post-Irene (8/31) minus pre-Irene (8/26) with NHC best track (black dots connected by dashed line labelled with August date and UTC time), weather buoys/stations (coloured diamonds), underwater glider RU16 location during storm (yellow square) and bathymetry at 50 m (dotted magenta) and 200 m (solid magenta). (**b**–**d**) Buoy/station observed SST (blue) and air temperature (red) with vertical black dashed line/label indicating the time/value of minimum air pressure (**b**,**c**), and time of eye passage according to NHC best track data (**d**). The individual SST three-day composite maps for 24–26 August and 29–31 August are provided in Supplementary Fig. 1A,B.

Hurricane Irene formed east of the Caribbean's Windward Islands on 22 August 2011 and made initial United States landfall in North Carolina as a Category 1 hurricane on 27 August. It re-emerged over the ocean in the MAB before a second landfall in New Jersey as a tropical storm on 28 August (ref. 5), closely following the historical northeastward tracks of hurricanes along the northeast United States^19^. Irene accelerated and lost intensity as it crossed the MAB, moving parallel to the coast with the eye over inner-continental shelf waters (Fig. 1a). Propagation was rapid at 30–40 km h^−1^, requiring only ∼9.5 h to cross from North Carolina to New Jersey landfall. Cloud bands extended over 600 km from the eye centre, obscuring the ocean from satellite infrared SST sensors during passage. Differencing 3-day composites of cloud-free satellite imagery before (24–26 August) from after (29–31 August) Irene reveals the regional pattern of MAB sea surface cooling (Fig. 1a and Supplementary Fig. 1A,B). The largest cooling (5–11 °C) was observed to the right of the eye centre over the MAB's middle to outer shelf. Inner shelf cooling was slightly less, with averages of 3–5 °C of cooling within the 25-km radius eye wall (Supplementary Fig. 1C). Cooling was much less significant on the shelf seas to the south of the MAB, in the deep ocean to the east and, as previously noted in other hurricanes^20^, along the very shallow unstratified coast, bays and sounds.

### Observations

National Data Buoy Center (NDBC) buoys 44009 and 44065 recorded peak wind speeds (Supplementary Fig. 2) near 20 m s^−1^ from offshore as Irene approached. At these NDBC buoys and at 44100, water temperatures dropped rapidly by 3.8–6.3 °C ahead of eye centre passage (Fig. 1b–d), representing 82–98% of the in-storm cooling at these locations (Supplementary Fig. 3). At Irene's fast propagation speed, the eye was still 150–200 km to the south after the most rapid cooling was complete. As the ocean surface cooled, observed air temperatures were greater than SSTs, indicating air–sea-sensible heat fluxes were from the atmosphere into the ocean.

**Figure 2:**
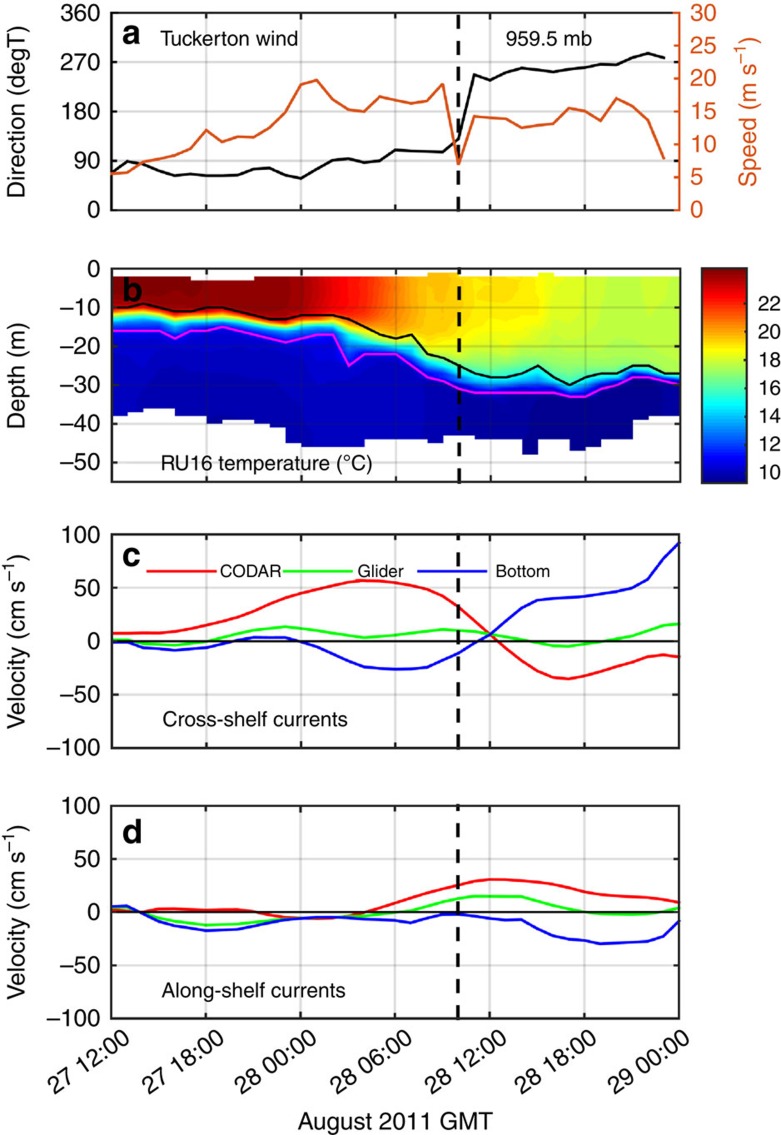
Data from a local meteorological station, glider and HF radar. (**a**) Tuckerton WeatherFlow, Inc. station 10 m wind speed (orange) and direction from (black) with vertical black dashed line/label indicating the time/value of the minimum air pressure corresponding to landfall time on 28 August at 935 GMT. (**b**) Temporal evolution and vertical structure of the glider temperature during storm conditions with lines indicating top (black) and bottom (magenta) of thermocline. (**c**) Cross-shelf currents (positive onshore, negative offshore) for the surface layer (red) from CODAR HF Radar, depth-averaged (green) from the glider and bottom layer (blue) calculated from the depth-weighted average of the HF radar and glider velocities. (**d**) Same as **c** but for along-shelf currents (positive up-shelf northeastward and negative down-shelf southwestward).

Atmospheric conditions (Fig. 2a) were recorded just inshore of a Slocum autonomous underwater glider^21^,^22^ measuring subsurface ocean conditions^23^ during Irene at the location shown in Fig. 1a (see Supplementary Fig. 4 for a plot of the complete glider track well before, during and after the storm). Winds initially from offshore (90°), with speeds near 20 m s^−1^ ahead of the eye, rotated rapidly to blow from onshore (270°) after the eye passed. Glider-observed subsurface temperatures (Fig. 2b) indicate that initially, typical MAB summer stratification^24^ was present, with a seasonally warmed surface layer (∼24 °C) above the MAB Cold Pool^25^ (<10 °C) separated by a sharp (<8 m thick) thermocline. Significant cooling of the surface layer (5.1 °C) and deepening of the thermocline (>15 m) was observed under the leading edge of the storm. Little change in thermocline depth and much less cooling (1.6 °C) of the upper layer was observed after eye passage. Thus, ahead-of-eye-centre cooling represents 76% of in-storm cooling observed at the glider (Fig. 2b). Both the glider and buoy data suggest that much of the satellite observed SST cooling (over ∼100,000 km^2^ of continental shelf) occurred ahead-of-eye-centre.

Ocean surface currents measured by a CODAR high-frequency (HF) radar^26^ network^27^ illustrated the rapid response of the thin surface layer (Supplementary Fig. 5) to the changing wind direction (Fig. 2a). Time-series of the cross-shelf components of the currents (Fig. 2c) at the glider location, with positive values towards land, indicate that the onshore surface currents began building before the eye entered the MAB, increasing to a peak value >50 cm s^−1^ towards the coast before the eye passage. Along-shelf currents throughout the water column were weak (Fig. 2d). After the eye, the winds changed direction and within a few hours, the cross-shelf surface currents switched to offshore. Despite the strong observed surface currents, the depth-averaged current (Fig. 2c) reported by the glider remained small during the storm's duration, with peaks barely exceeding 5 cm s^−1^. As in deep water, the current response is baroclinic^28^,^29^, but the low depth-averaged current implies a strong offshore flow in the bottom layer. These bottom layer currents were estimated based on the relative layer thicknesses and the requirement that the combined surface and bottom layer-averaged currents matched the glider-observed dead-reckoned depth-averaged current. The estimated bottom layer currents accelerated in the offshore direction as the eye approached, causing significant shear between the two layers at the same time the surface layer was deepening and cooling.

### Ocean model simulations

**Figure 3:**
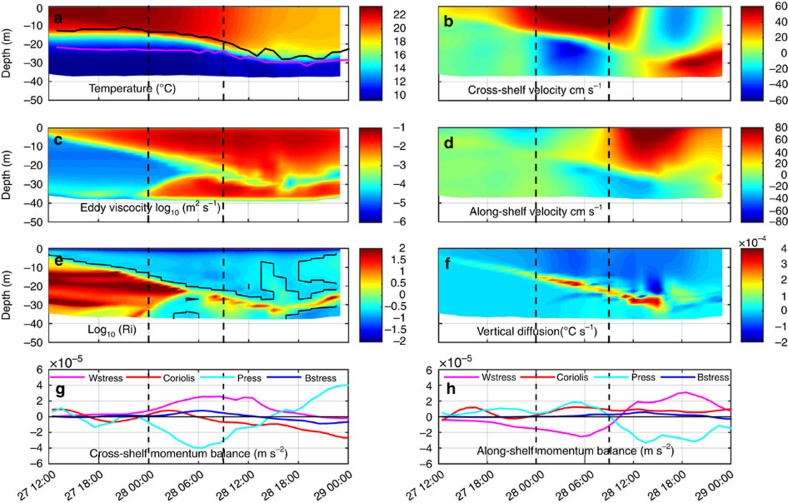
ROMS ocean simulation results at the glider location. ROMS ocean simulation results at the glider location during the storm period, with first vertical black dashed line indicating initiation of the coastal baroclinic response and second vertical black dashed line indicating eye passage. (**a**) Temperature with top (black) and bottom (magenta) of thermocline as in Fig. 2b. (**b**) Cross-shelf velocity (red/yellow onshore; blue offshore). (**c**) Eddy viscosity. (**d**) Along-shelf velocity (red/yellow northward; blue southward). (**e**) Log_10_(Richardson number) with black contour indicating Richardson number of 0.25. (**f**) Vertical diffusion temperature diagnostic equation term, showing warming (positive, red/yellow) and cooling (negative, dark blue). (**g**) Dominant depth-averaged cross-shelf momentum balance terms (positive onshore and negative offshore) from wind stress (wstress, magenta), Coriolis force (coriolis, red), pressure gradient (press, cyan) and bottom stress (bstress, blue). (**h**) Same as **g** but for along-shelf momentum balance terms (positive northward, negative southward).

Coastal ocean three-dimensional (3D) model simulations of Irene using the Regional Ocean Modeling System (ROMS) in the MAB^30^,^31^ successfully reproduced the thermocline deepening and surface layer cooling (Fig. 3a) similar to the glider observations (Fig. 2b). The modelled cross-shelf velocity component (Fig. 3b) also has similarities to the combined glider and HF radar data (Fig. 2c). The surface layer flow accelerated shoreward for 12 h until eye passage, while the bottom layer responded more slowly with an offshore counter-flow. A few hours after eye passage, the cross-shelf flows reversed, also consistent with observations. The dominant terms in the cross-shelf momentum balance (Fig. 3g) indicate that the surface wind stress increased as the eye approached and decreased as it receded. Before the eye centre arrival, the presence of a coastline produced an offshore-directed pressure gradient that nearly balanced the wind stress and accelerated the offshore jet in the bottom layer. After the storm passage, the cross-shelf surface current switched to offshore; the cross-shelf pressure gradient also switched sign and was redirected towards the coast. At this point in the storm, the dominant cross-shelf momentum balance was nearly geostrophic (Fig. 3g) with a northward along-shelf surface current (Fig. 3d).

The subsurface cross-shelf circulation within the two-layer coastal ocean had a significant influence on vertical mixing as illustrated by the Richardson number (Fig. 3e) and the vertical eddy viscosity (Fig. 3c). The Richardson number and the eddy viscosity show that the surface layer deepened to meet the stratification at the top of the thermocline as the surface layer accelerated with the approaching storm. As the offshore counter current accelerated in the bottom boundary layer, the lower layer Richardson number also decreased and eddy viscosity increased until the two layers interacted. The most rapid ahead-of-eye-centre cooling and deepening of the surface layer occurred when the small Richardson numbers and large vertical eddy viscosities from the surface and bottom boundary layers overlapped. The model's temperature diagnostic equation indicates that vertical diffusion (Fig. 3f) was the dominant term (Supplementary Fig. 6) acting to deepen the thermocline and cool the surface layer during the event.

### Atmospheric model simulations

**Figure 4:**
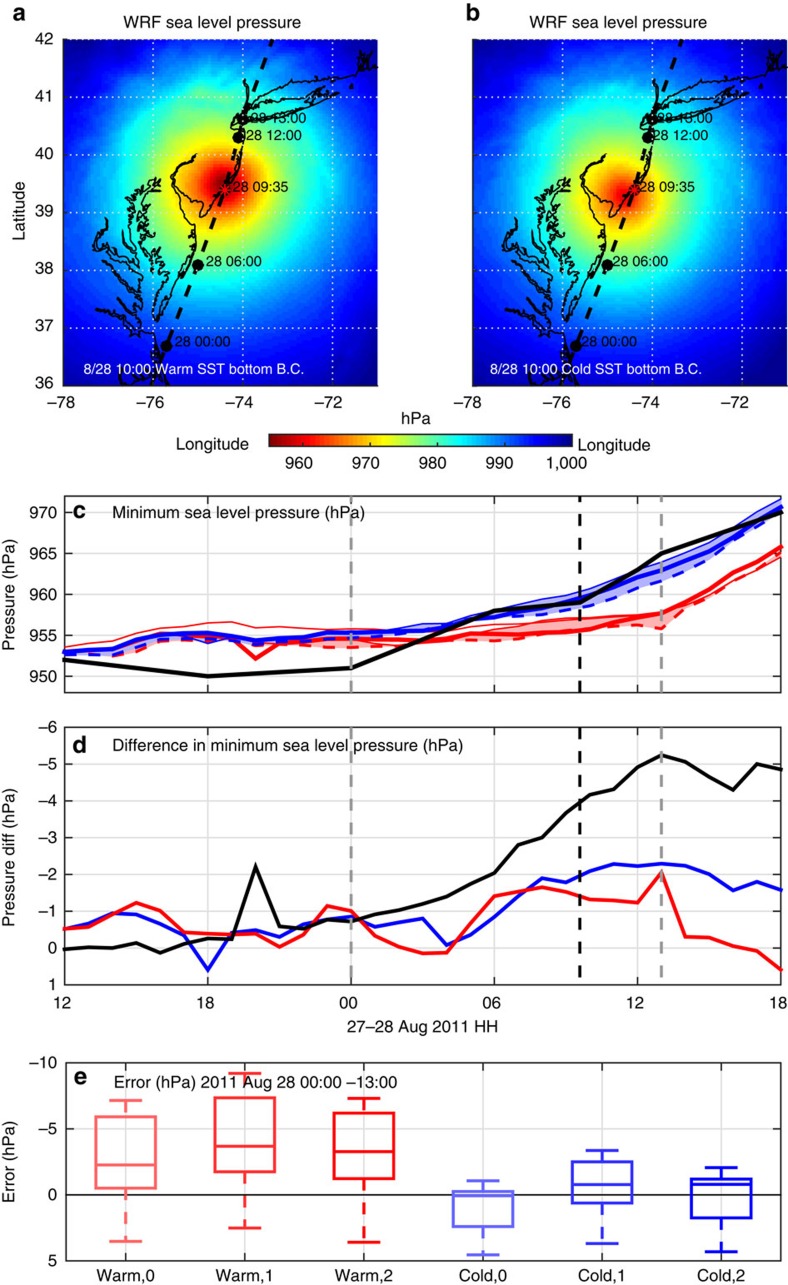
WRF atmospheric model simulation results. (**a**) WRF model SLP (with surface flux option 2) at landfall (red star) for the warm SST boundary condition with NHC best track drawn as in Fig. 1a. (**b**) Same as **a** but for the cold SST. (**c**) Minimum SLP for NHC best track (black), and WRF's three air–sea flux parameterization options isftcflx=0 (thin line); 1 (dotted line); and 2 (thick line) for the warm (red) and cold (blue) SST. Vertical grey and black dashed lines indicate eye enters MAB, makes landfall and leaves MAB. (**d**) Model SLP sensitivity to SST (black, warm minus cold SST for isftcflx=2), and to flux parameterizations (isftcflx=1 minus isftcflx=0) for warm (red) and cold (blue) SST. (**e**) Box and whisker plots of SLP deviations from NHC best track when eye is over MAB for warm (red) and cold (blue) SST.

Atmospheric model simulations of Irene used the Weather Research and Forecasting (WRF)^32^ model as applied to the US East Coast for tropical cyclone forecasting^33^. Typical surface boundary approaches in uncoupled atmospheric models use satellite SSTs over water that remain fixed when new data is not available because of cloud cover. A matrix of over 130 simulations revealed ahead-of-eye-centre cooling of the ocean's surface layer has a significant impact on intensity as reflected in the hurricane pressure (Fig. 4) and wind fields (Supplementary Fig. 7). Examining the ensemble of simulations with track errors less than one eye-wall radius, the largest wind and pressure intensity sensitivities were generated using fixed warm pre-storm and cold post-storm SST boundary conditions (Supplementary Figs 8,9). The sea level pressure (SLP) fields at landfall indicate the warm (Fig. 4a) versus the cold (Fig. 4b) SST changed the centre SLP by 7–8 hPa, with the maximum wind speed reduced by >5 m s^−1^ due to the cooler SST (Supplementary Fig. 7). The minimum SLP time history (Fig. 4c) of selected model runs can be compared with the National Hurricane Center (NHC) best track parameters. The best track central pressure remains constant near 952 hPa until the eye enters the MAB (28 August at about 00 h), followed by a steady increase in the central pressure to 965 hPa 13 h later as the eye leaves the MAB. Once Irene's eye entered the MAB, the cold SST air–sea flux parameterization sensitivities all produce a reduction in intensity that cluster with the best track analysis, while the warm SST air–sea flux parameterization sensitivities maintain a lower minimum SLP with little change nearly until landfall.

The top three model sensitivities are quantified by the envelope width for the minimum SLP (Fig. 4d). For both warm and cold SSTs, sensitivities to the three standard WRF air–sea flux formulations range from 0 to 2 hPa for the 13 h after the eye entered the MAB. The sensitivity to warm and cold SST begins growing as the storm nears the MAB, climbing steadily to 5 hPa as it leaves the MAB. Statistical comparisons of each model run to the NHC best track over the MAB are quantified by the box and whisker plots (Fig. 4e) showing the median, inter-quartile range and outliers. The three warm SST air–sea flux sensitivities consistently over-predict the intensity with minimum SLPs that are too low, while the three cold SST air–sea flux sensitivities more accurately reflect the intensity reduction for all of the air–sea flux options.

## Discussion

Using Hurricane Irene as a diagnostic case study, a new feedback mechanism on storm intensity in the coastal ocean has been identified. The strong onshore winds occurring ahead-of-eye-centre in tropical cyclones and the coastal wall set up a down-welling circulation that limits the storm surge and results in significant shear across the thermocline. This shear leads to turbulent entrainment of abundant cold bottom water and mixing with warmer surface water. The resulting ocean cooling reduces surface heat fluxes to the atmosphere, weakening the storm.

Rapid tropical cyclone intensity changes over the deep ocean have been correlated with storm passage over warm and cold core eddies^12^,^13^,^14^,^15^,^16^,^34^. Also in the deep ocean, SST changes of as little as 1 °C are noted to significantly impact storm intensity^9^,^35^. During Hurricane Irene, ahead-of-eye-centre cooling of 3.8–6.3 °C was observed with nearshore buoys (Supplementary Fig. 3) and 5.1 °C was observed with a mid-shelf glider (Fig. 2). Storm-induced cooling in deep water is often equally distributed between the front and back half of the storm^36^. Deep ocean simulations of Irene with both a 1D ocean mixed layer model and the 3D Price–Weller–Pinkel^37^ model produced 32 and 56% of the in-storm cooling ahead-of-eye-centre, respectively. In Hurricane Irene, 76% (glider) to 98% (buoy 44100) of the in-storm cooling occurred ahead-of-eye-centre, indicating that coastal baroclinic processes are enhancing the percentage of ahead-of-eye-centre cooling in Irene.

To verify that enhanced ahead-of-eye-centre coastal ocean cooling is not unique to Irene, 30 years of historical nearshore buoy data throughout the MAB were investigated. During that time period, ahead-of-eye-centre cooling was observed in all 11 tropical cyclones that tracked northeastward over the MAB continental shelf during the highly stratified summer months (June–August)^24^,^38^ (Table 1 and Supplementary Figs 10–12). The maximum continental shelf buoy observed ahead-of-eye-centre cooling for these 11 storms averages 2.7±1.3 °C, representing an average of 73% of the in-storm cooling.

An 11-year global satellite climatology^39^ reveals that the shallow mid-latitude Yellow Sea and northern East China Sea also experience a large 20 °C seasonal SST cycle, similar to the MAB but over three times larger in area. A 1986 Yellow Sea shipboard conductivity temperature and depth survey reports surface to bottom temperature differences approaching 15 °C (ref. 40), also similar to the stratified summer MAB. Maps of western Pacific typhoon tracks ( coast.noaa.gov/hurricanes) indicate 26 typhoons have tracked across the northern East China Sea and Yellow Sea during June–August since 1985. Like Irene, the landfalling intensity of Super Typhoon Muifa (2011) was over-predicted by standard models^41^. Satellite SST maps indicate Muifa caused significant in-storm cooling (up to 7 °C) across ∼300,000 km^2^ of the continental shelf^41^. Nearshore buoy observations show cooling of 4.1 °C (85% of the in-storm cooling observed at that location) was ahead-of-eye-centre (Table 1, Supplementary Fig. 13).

Globally, over the past 30 years, tropical cyclone maximum intensities have migrated poleward^42^. In the North Atlantic, hurricane intensities have increased since the early 1980s and are projected to continue to increase as the climate warms^43^,^44^,^45^,^46^. Combined with rapid sea level rise^47^, mid-latitude population centres will experience heightened vulnerability to storm surge and inundation from increasingly powerful storms. To mitigate these risks, improved forecasting of tropical cyclone intensity over mid-latitude stratified coastal seas is vital, and will require realistic 3D ocean models to forecast enhanced ahead-of-eye-centre cooling.

## Methods

### Data source

The Mid-Atlantic Regional Association Coastal Ocean Observing System (MARACOOS) is a sustained regional component of the US Integrated Ocean Observing System (IOOS)^48^. Its integrated observation network of satellites, buoys, coastal meteorological stations, HF radar and autonomous underwater gliders provided the data used in this study^49^.

### Satellite remote sensing

National Oceanographic and Atmospheric Administration (NOAA) Advanced Very High-Resolution Radiometer (AVHRR) satellite data (Supplementary Fig. 1) were acquired through a SeaSpace TeraScan L-Band satellite ground station at Rutgers University. AVHRR data are converted to SST using the multi-channel SST algorithm^50^. To specifically map areas of rapid cooling, a ‘coldest-dark-pixel' composite technique is used to identify and remove bright cloud covered pixels while retaining the darker ocean pixels. This is accomplished through the following series of tests performed on AVHRR channels 4 and 2 scans. Pixels are considered contaminated by clouds and removed if (1) AVHRR channel 4 (10.3–11.3 μm) temperatures are <5 °C (3.5 °C) in summer (winter); or (2) near infrared albedo in daytime AVHRR Channel 2 (0.725–1 μm) exceeds 2.3% (an empirically derived threshold specific to the MAB). Further tests are performed on 3 × 3 km grid boxes to account for large changes in temperature over short distances typical of cloud edges. Centre pixels are flagged as potential cloud edges and removed if (1) temperature changes in AVHRR channel 4 scans are >1 °C across the centre point of each 3 × 3 grid data; or (2) the change in infrared albedo across the centre of each 3 × 3 grid box is >0.15%. After declouding is performed, the resulting 3 days of scans between 12:00 to 17:00 GMT are composited with the NASA (National Aeronautics and Space Administration) short-term Prediction Research and Transition centre (SPoRT) 2 km blended 7-day SST product. At each pixel the coldest value is retained between all daytime AVHRR scans for the past 3 days and the SPoRT SST product for that day to ensure retention of coastal upwelling zones and regions that underwent rapid mixing. Consistent with real-time processing protocols, the date assigned to each composite corresponds to the final day of the data window.

### Meteorological observations

Meteorological observations were obtained from NOAA NDBC buoys, coastal towers and pier stations, and a WeatherFlow Inc. meteorological tower located in Tuckerton, New Jersey (Fig. 1a). Buoys 44009 (38.461° North and 74.703° West) and 44065 (40.369° North and 73.703° West) included wind speed and direction measured at a height of 5 m, air temperature at a height of 4 m and ocean temperatures at 0.6 m depth. Buoy 44100 (36.255° North and 75.591° West) is a Waverider buoy managed by Scripps Institution of Oceanography that measured ocean temperatures at 0.46 m depth. Station DUKN7 (36.184° North and 75.746° West) is a coastal station that measures air temperature at 15.68 m above mean sea level. The Tuckerton WeatherFlow Inc. meteorological tower (39.52° North and 74.32° West) measured wind speed and direction at 12 m. Meteorological data is plotted at the standard frequencies and averaging intervals reported by these stations.

### High frequency radar

A network of over 40 CODAR Ocean Sensors SeaSonde HF Radar stations^26^ are deployed along the MAB coast by a consortium of institutions coordinated through MARACOOS^27^. The stations transmit HF radio waves that are scattered off the ocean surface waves and then received back on shore. The Doppler shift in the Bragg peaks of the received signal are used to map the radial components of the total surface velocity field in front of each station^51^. Radial components from multiple stations are combined using an optimal interpolation scheme^52^ to produce 1 h centre-averaged hourly surface current maps^53^ with a nominal 6 km spatial resolution (Supplementary Fig. 5).

### Autonomous underwater gliders

Teledyne Webb Research Slocum gliders are buoyancy-driven underwater vehicles that act as mobile sensor platforms^22^. These instrument platforms adjust small amounts of buoyancy in order to glide through the water column at 20–30 cm s^−1^ in a sawtooth pattern. At pre-programmed intervals the gliders come to the surface and transfer data back to Rutgers University in near real-time. The glider used in this study, RU16, was equipped with an un-pumped Seabird conductivity temperature and depth sensor that logged data every 4 s on downcasts and upcasts. Depth- and time-averaged velocity calculations were performed using a dead-reckoning technique typical for such platforms^22^,^54^,^55^. The measured pitch angle, fall velocity and a model of glider flight to estimate angle of attack are used to calculate an underwater horizontal displacement during each dive segment. The difference between the calculated horizontal displacement from the final pre-dive location and the actual surfacing location divided by the time underwater provides an estimate of depth- and time-averaged velocity.

A combination of dead-reckoned depth-averaged glider currents and HF radar surface currents are used to estimate bottom currents along the glider track (Fig. 2c). The following algorithm assumes that the HF radar surface currents are representative of the surface layer above the thermocline (defined as the maximum vertical temperature gradient along each profile) and requires that the depth-weighted average surface and bottom layer currents must equal the total depth-averaged glider current:


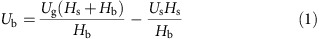



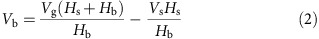


where *H*_s_ and *H*_b_ are the layer thicknesses above and below the thermocline, respectively, *U*_g_ and *V*_g_ are along- and cross-shelf depth-averaged currents, respectively, from glider dead-reckoning, *U*_s_ and *V*_s_ are surface layer-averaged currents from HF radar, and *U*_b_ and *V*_b_ are the calculated bottom layer-averaged currents (Fig. 2).

### ROMS model setup

The numerical simulations were conducted using the ROMS^31^, a free-surface, sigma coordinate, primitive equation ocean model (code available at http://www.myroms.org) that has been widely used in a diverse range of coastal applications. The ESPreSSO (Experimental System for Predicting Shelf and Slope Optics) model^56^ covers the MAB from the centre of Cape Cod southward to Cape Hatteras, from the coast to beyond the shelf break and shelf/slope front. Gridded bathymetric data is used to construct a model grid with a horizontal resolution of 5 km (Supplementary Fig. 4) and 36 vertical levels in a terrain-following s-coordinate system. The initial conditions were developed from the same domain ROMS run with strong constrained four-dimensional variational (4D-Var) data assimilation^57^. The meteorological forcing is from the North American Mesoscale (NAM) model 12 km 3-hourly forecast data. Reanalyses of surface air temperature, pressure, relative humidity, 10 m vector winds, precipitation, downward longwave radiation and net shortwave radiation were used to specify the surface fluxes of momentum and buoyancy based on the COARE bulk formulae^58^. Boundary conditions are daily two-dimensional surface elevation, as well as three-dimensional velocity, temperature, and salinity fields from the Hybrid Coordinate Ocean Model Navy Coupled Ocean Data Assimilation forecast system. Inflows for the seven largest rivers are from daily average United States Geological Survey discharge data. Tidal boundary conditions are from the The ADvanced CIRCulation tidal model. The general length scale method k-kl type vertical mixing scheme^59^,^60^ is used to compute vertical turbulence diffusivity.

### ROMS momentum balance analysis

We extracted depth-averaged momentum balance terms from ROMS (Fig. 3g–h) at the glider sampling location in order to diagnose the dominant forces during the storm, where the acceleration terms are balanced by a combination of horizontal advection, pressure gradient, surface and bottom stresses and the Coriolis force (horizontal diffusion was small and neglected in this case):


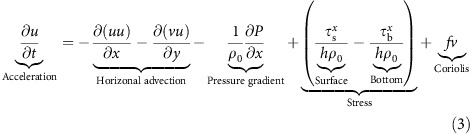



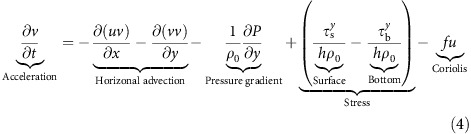


where *u* and *v* are the along-shelf and cross-shelf components of velocity respectively, *t* is time, *P* is pressure, *ρ*_*o*_ is a reference density, *τ*_s_ and *τ*_b_ are surface and bottom stresses, *h* is water column depth and *f* is the latitude-dependent Coriolis frequency.

### ROMS heat balance analysis

*Heat balance analysis*. The general conservation expression for the temperature budget in ROMS is given by


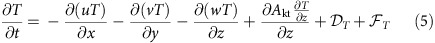


with the following surface and bottom boundary conditions:


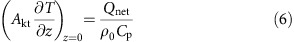



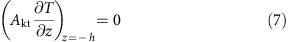


Here, *T* is the temperature, *t* is time, *u*, *v* and *w* are the along-shelf, cross-shelf and vertical components of velocity. *A*_kt_ is the vertical diffusivity coefficient, 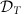 is the horizontal diffusion term and 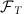 is friction. *Q*_net_ is the surface net heat flux, *ρ*_0_=1025, kg m^−3^ is a reference density, *C*_p_=3985 J (kg °C)^−1^ is the specific heat capacity of seawater and *h* is the water depth.

The ROMS conservation of heat equation was used to diagnose the relative contributions of the different terms responsible for the modelled temperature change. Time-series of the vertical temperature diagnostic terms were investigated along the glider track with emphasis on the temperature evolution between the top of the thermocline depth (the shallowest location where the vertical temperature gradient exceeded 0.4 °C m^−1^, black contour in Fig. 3a and Supplementary Fig. 6) and the transition layer depth (the deepest location where the vertical temperature gradient exceeded 0.7 °C m^−1^, magenta contour in Fig. 3a and Supplementary Fig. 6). Term-by-term analysis of equation 5 offered additional insights on the temperature source and sink terms. Supplementary Fig. 6A shows the temperature rate of change, which is the sum of the vertical diffusion term (Supplementary Fig. 6B) and advection term (Supplementary Fig. 6C), in which the advection term is separated into along-shelf advection (Supplementary Fig. 6D), cross-shelf advection (Supplementary Fig. 6E) and vertical advection (Supplementary Fig. 6F). The horizontal diffusion term's order of magnitude is much smaller than other terms and is not plotted. The dominant term influencing the surface mixed layer temperature change was the vertical diffusion, which is plotted in Fig. 3f.

### WRF-ARW model setup

The Weather Research and Forecasting Advanced Research (WRF-ARW) dynamical core (code available at http://www.wrf-model.org)^32^, Version 3.4 was used for the atmospheric simulations in this study. WRF-ARW is a fully compressible, non-hydrostatic, terrain-following coordinate, primitive equation atmospheric model. Our WRF-ARW domain extends from South Florida to Nova Scotia (Supplementary Fig. 14), with grid resolution of 6 km in the horizontal and 35 vertical levels. Lateral boundary conditions used are from the Global Forecast System (GFS) 0.5° initialized at 06 UTC on 27 August 2011.

Our simulations begin at 06 UTC on 27 August 2011 when Hurricane Irene was south of North Carolina (NC) over the South-Atlantic Bight (SAB) and end at 18 UTC on 28 August 2011 as the storm moved into New England. Simulation results shown (Fig. 4c,d and Supplementary Fig. 7C,D) begin at 12 UTC on 27 August 2011, at NC landfall time, after the model has 6 h to adjust to vortex initialization. WRF's digital filter initialization (DFI) was run to determine the sensitivities to different realizations of the GFS initializations. DFI deepened the initial vortex central pressure by over 10–960 hPa, which matches GFS initial central pressure (Supplementary Fig. 15). However, downstream sensitivity to DFI beyond 2 h was minimal.

For our control run, the following are used: longwave and shortwave radiation physics were both computed by the Rapid Radiative Transfer Model-Global scheme; the Monin–Obukhov atmospheric layer model and the Noah Land Surface Model were used with the Yonsei University planetary boundary layer scheme; and the WRF Double-Moment 6-class moisture microphysics scheme was used for grid-scale precipitation processes.

### WRF sensitivity to SST

The model was run over 130 times to compare the sensitivity of certain parameter tuning. All sensitivities were compared to the control run (described above), which for surface boundary conditions over the ocean, that is, SST, used the Real-Time Global High-Resolution (RTG HR) SST analysis from 00 UTC on 27 August 2011 fixed throughout the simulation. This is the warm pre-storm SST, and has temperatures across the model domain similar to the AVHRR coldest-dark-pixel composite a day earlier (Supplementary Fig. 1A). By having the control run use Real-Time Global High-Resolution SST fixed throughout the simulation, we are consistent with what the operational NAM 12 km model used for bottom boundary conditions over the ocean.

To show the maximum impact of the ahead-of-eye-centre SST cooling on storm intensity, we compared our control run with a simulation using observed cold post-storm SST. For this, we used our AVHRR coldest-dark-pixel composite, which includes data from 29 to 31 August 2011 (Supplementary Fig. 1B). According to underwater glider and NDBC buoy observations along Irene's entire MAB track, almost all of the SST cooling occurred ahead of Irene's eye centre (Fig. 1b–d). NDBC buoy observations near Irene's track in the SAB (41013, 41036, 41037) also show ahead-of-eye-centre SST cooling, but values are on the order of 1 °C or less (Fig. 1a). Because our model simulations include only 6 h of storm presence over the SAB before NC landfall, and SST cooling in the SAB was significantly less than observed in the MAB (Fig. 1), we can conclude that the main result from our SST sensitivity is due to the ahead-of-eye-centre cooling in the MAB.

### WRF sensitivity to air-sea flux parameterizations

The equations for the momentum (τ), sensible (*H*) and latent heat fluxes (*E*) are as follows:


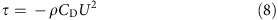



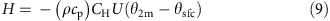



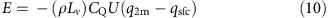


where *ρ* is density of air, *C*_D_ is drag coefficient, *U* is 10 m wind speed, *c*_p_ is specific heat capacity of air, *C*_H_ is sensible heat coefficient, *θ*_*2*m_ is potential temperature at 2 m and *θ*_sfc_ is potential temperature at the surface, *L*_*ν*_ is enthalpy of vaporization, *C*_*Q*_ is latent heat coefficient, *q*_2m_ is specific humidity at 2 m and *q*_sfc_ is interfacial specific humidity at the surface.

Three options exist in WRF-ARW Version 3.0 and later for air–sea flux parameterizations (WRF namelist option isftcflx=0, 1, and 2; see (ref. 61) for more details). These parameterization options change the momentum (*z*_0_), sensible heat (*z*_T_) and latent heat roughness lengths (*z*_Q_) in the following equations for drag (*C_D_*), sensible heat (*C_H_*) and latent heat (*C_Q_*) coefficients:


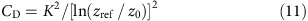



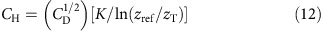



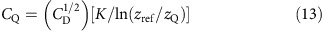


where *K* is the von Kármán constant and *z*_ref_ is a reference height (usually 10 m).

Therefore, our SST sensitivity effectively changes the variables *θ*_sfc_ and *q*_sfc_ in equations 8, 9, 10 above, while our air–sea flux parameterization sensitivities change the equations for the momentum, sensible heat and latent heat coefficients (equations 11, 12, 13) going into the respective flux equations 8, 9, 10.

For our air–sea flux parameterization sensitivities in this study, we ran isftcflx=0, 1, and 2 with both the warm (control) and cold SST boundary conditions.

### Additional WRF sensitivities

We have discussed SST and air–sea flux parameterizations. WRF-ARW was run over 130 times in total, with various model configuration and physics options turned on and off.

We examined the ensemble of simulations with space/time track errors <25 km (one eye-wall radius) from available NHC best track positional data. Only preserving those simulations with accurate tracks is important because Hurricane Irene tracked close to and parallel to the Mid-Atlantic coast. The remaining sensitivities are shown in central pressure (Supplementary Fig. 8) and maximum winds (Supplementary Fig. 9). These are cumulative hourly sensitivities during Irene's presence over the MAB and NY Harbor (28 August 00-13 UTC). Supplementary Table 1 shows a list of these sensitivities, with the WRF namelist option number alongside its name (control run listed last for each sensitivity).

The sensitivity titled ‘latent heat flux <0 over water' requires a brief explanation. In the WRF surface layer scheme code, there is a switch that disallows any latent heat flux less than 0 W m^−2^ (similarly, there is a switch that disallows any sensible heat flux less than −250 W m^−2^). WRF convention for negative heat flux is downward, or atmosphere to land/water. We run WRF after removing the line of code disallowing negative latent heat flux, and compare to the control run. This switch removal only changes latent heat flux and allows it to be negative over water, as the subsequent WRF land surface scheme modifies fluxes and allows for negative latent heat flux over land.

### Ahead-of-eye-centre and in-storm cooling calculations

Ahead-of-eye-centre cooling (Table 1) at NDBC buoys (Supplementary Figs 10–12) and the Yellow Sea buoy (Supplementary Fig. 13) was calculated by taking the difference between the maximum water temperature as the winds increased above 5 m s^−1^ and the minimum water temperature before or at the minimum observed SLP. In-storm cooling was determined as the difference between the same maximum water temperature as the winds increased above 5 m s^−1^ and the minimum water temperature while winds remained above 5 m s^−1^ after the pressure minimum. To calculate the average and standard deviation of cooling for the 11 storms passing through the MAB since 1985, we selected the one buoy on the continental shelf that recorded wind speed, pressure and water temperature and exhibited the greatest ahead-of-eye-centre cooling. For completeness we show Irene cooling statistics (Table 1) and time-series (Supplementary Fig. 3) for buoys 44065 and 44100 used in Fig. 1.

### Data availability

Buoy meteorological data used in this study are available through the National Data Buoy Center. Glider and HF Radar data can be found through the MARACOOS THREDDS server at http://maracoos.org/data. Tuckerton meteorological data are supported by WeatherFlow Inc. and can be made available upon request to the corresponding authors. WRF and ROMS model simulations are stored locally at the Rutgers Department of Marine and Coastal Sciences and will be made available upon request to the corresponding authors. The Yellow Sea buoy data are stored at the Institute of Oceanology, Chinese Academy of Sciences.

## Additional information

**How to cite this article:** Glenn, S. M. *et al*. Stratified coastal ocean interactions with tropical cyclones. *Nat. Commun.* 7:10887 doi: 10.1038/ncomms10887 (2016).

## Supplementary Material

Supplementary Information Supplementary Figures 1-15 and Supplementary Table 1

## Figures and Tables

**Table 1:** Sea surface temperature cooling in coastal tropical cyclones.

Storm name	Buoy	Water depth (m)	Ahead-of-eye-centre cooling (°C)	In-storm cooling (°C)	% Ahead-of-eye-centre
Arthur (2014)	44014	48	1.4	2.4	58%
Irene (2011)	44009	26	4.5	5.5	82%
Barry (2007)	ALSN6	29	5.1	5.1	100%
Hermine (2004)	44009	31	0.9	1.1	82%
Allison (2001)	CHLV2	14	2.3	2.6	88%
Bonnie (1998)	CHLV2	14	4.2	4.2	100%
Danny (1997)	44009	31	2.1	3.6	58%
Arthur (1996)	44009	31	2.3	3.5	66%
Emily (1993)	44014	48	2.3	2.8	82%
Bob (1991)	44025	41	2.1	4.6	46%
Charley (1986)	44009	31	2.7	5.4	50%
**Average**		**31**	**2.7**	**3.7**	**73%**
**Standard deviation**		**11**	**1.3**	**1.4**	**19%**
Irene (2011)	44065	25	3.8	4.2	90%
Irene (2011)	RU16	37–46	5.1	6.7	76%
Irene (2011)	44100	26	6.3	6.4	98%
Muifa (2011)	37.045 N 122.66 E	31	4.1	4.8	85%

Ahead-of-eye-centre cooling (°C), in-storm cooling (°C) and % ahead-of-eye-centre observed at nearshore MAB buoys for 11 tropical cyclones that traversed the MAB continental shelf during summer stratified conditions since 1985, additional data from Hurricane Irene and Super Typhoon Muifa.
