# Supplementary material for: Stratified coastal ocean interactions with tropical cyclones
Source: Nat Commun. 2016 Mar 8;7:10887. doi: 10.1038/ncomms10887 (PMC4786775; doi:10.1038/ncomms10887)
Supplement: Supplementary Information — Supplementary Figures 1-15 and Supplementary Table 1 [file ncomms10887-s1.pdf]

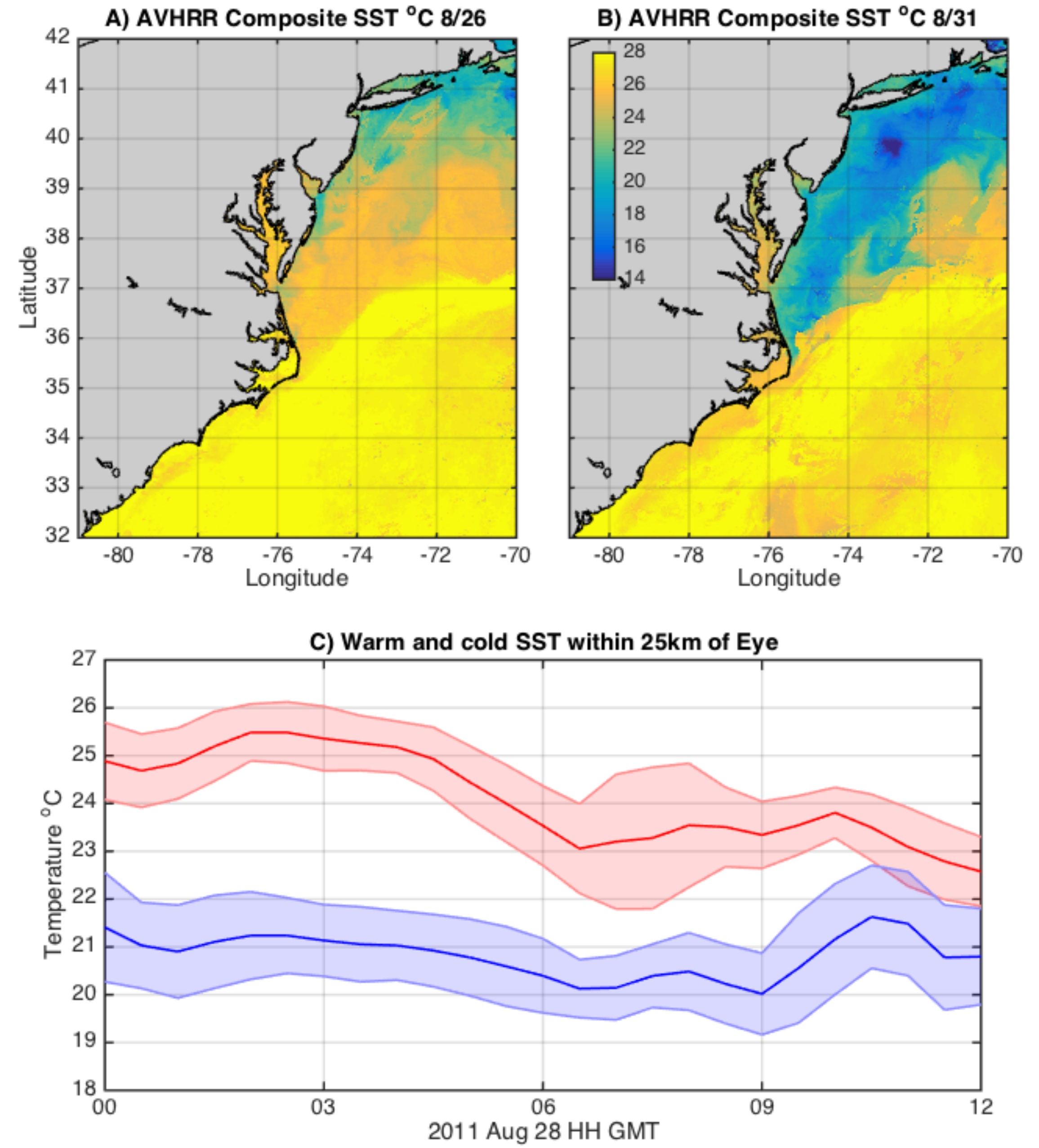

Supplementary Fig. 1 AVHRR coldest-dark-pixel three-day composite images from (A) August 24-26 2011 before Irene and (B) August 29-31 2011 after the storm. (C). The mean (solid lines) and standard deviations (shaded region) of temperatures within 25 kilometers of the NHC best track from the warm pre-storm AVHRR SST (A) and cold post-storm AVHRR SST (B). Landfall time was on August 28th 2011 at 9:35 GMT.

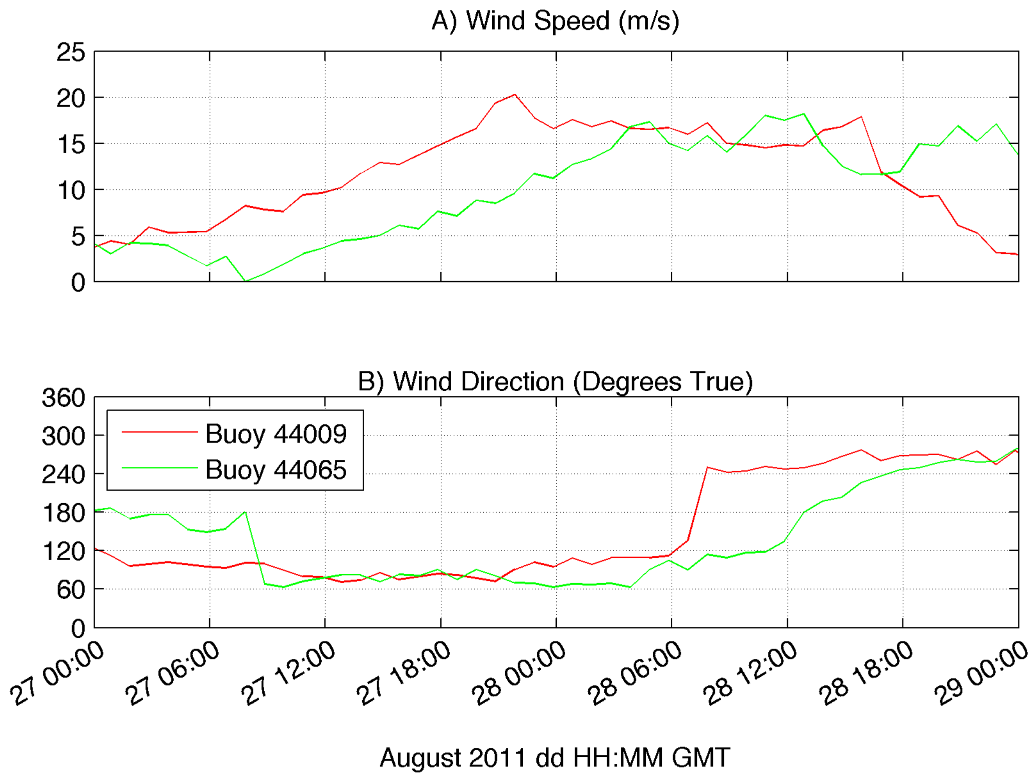

Supplementary Fig. 2 NDBC buoy 44009 and 44065 (A) hourly mean wind speed (m s^{-1}) and (B) wind direction (from degrees north).

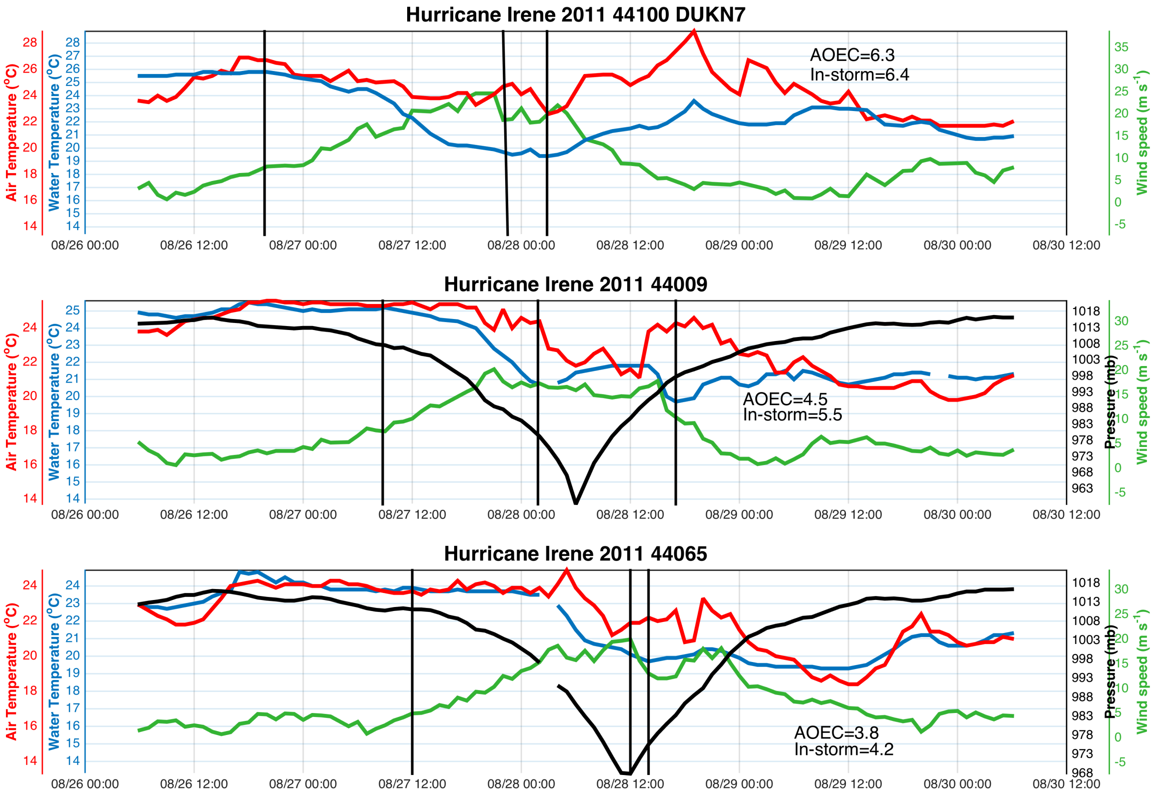

Supplementary Fig. 3 Time-series plots of air temperature (red), water temperature (blue), pressure (black), and wind speed (green). The first vertical black line indicates the maximum water temperature before wind speed increased above 5 $m\ s^{-1}$, the second represents the minimum temperature ahead-of or at the minimum pressure, and the third vertical black line is the minimum temperature before wind speeds drop below 5 $m\ s^{-1}$. For the top panel, air temperature and wind speed are from a nearby pier (DUKN7), water temperature is from 44100, and neither location had pressure. The NHC best track is used to estimate eye-passage time as August 27 at 2230 GMT 2011. Values from these plots are used to determine ahead-of-eye-center (AOEC) and in-storm cooling included in Table 1 for Hurricane Irene (2014).

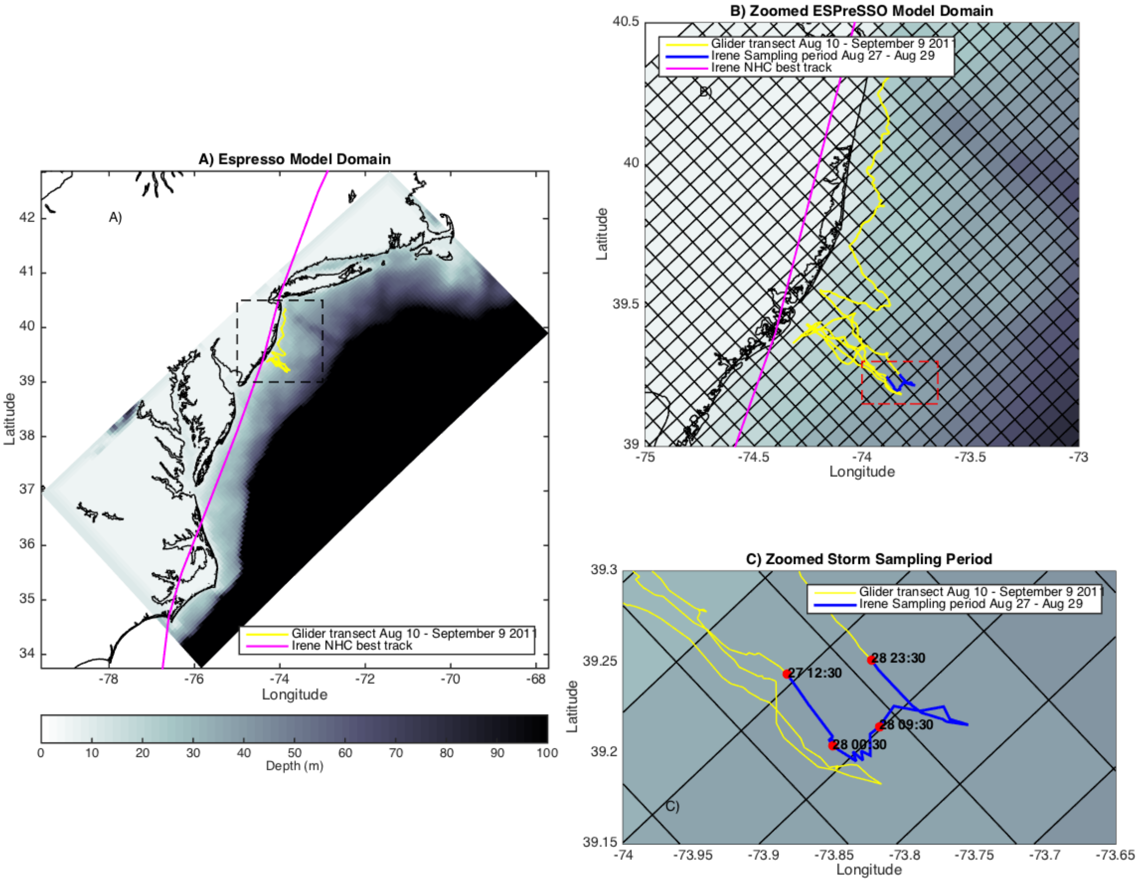

Supplementary Fig. 4 Plots of the glider deployment on the MAB with the shaded ESPreSSO ROMS bathymetry. Panel A) is the full ESPreSSO domain with the full glider track from August 10th to September 9th 2011 (yellow) and the NHC best track for Irene (magenta). Panel B) is a zoomed in view of the black dotted box region in A) showing the full glider transect (yellow), storm sampling period from August 27th to the 29th (blue) and NHC best track (magenta), with grid lines showing the ESPreSSO 5 km X 5 km model resolution relative to the glider. Panel C) is an even more zoomed in view of the red dashed box in B) showing a small portion of the full glider transect (yellow) and the storm sampling period (blue) from August 27 at 1200 GMT to August 28 at 2330 GMT with grid lines showing the ESPreSSO 5 km X 5 km model resolution relative to the glider. The glider remained in a small ~5km region during the entire storm sampling period, thus representing an Eulerian view of the time evolution of the coastal ocean during Irene.

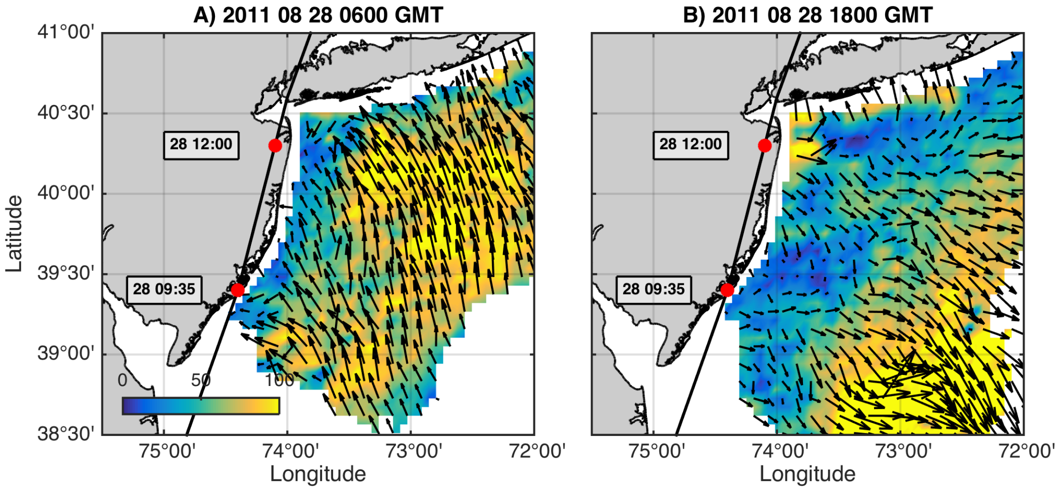

Supplementary Fig. 5 CODAR HF radar surface current maps in Hurricane Irene with vectors indicating direction and colors indicating current speeds (cm/s), approximately 4 hours before (A) and 8 hours after (B) landfall in New Jersey. The NHC best track for Irene is plotted in black with red dots indicating eye-position at landfall at 9:35 GMT on August 28th and post-landfall at 12:00 GMT.

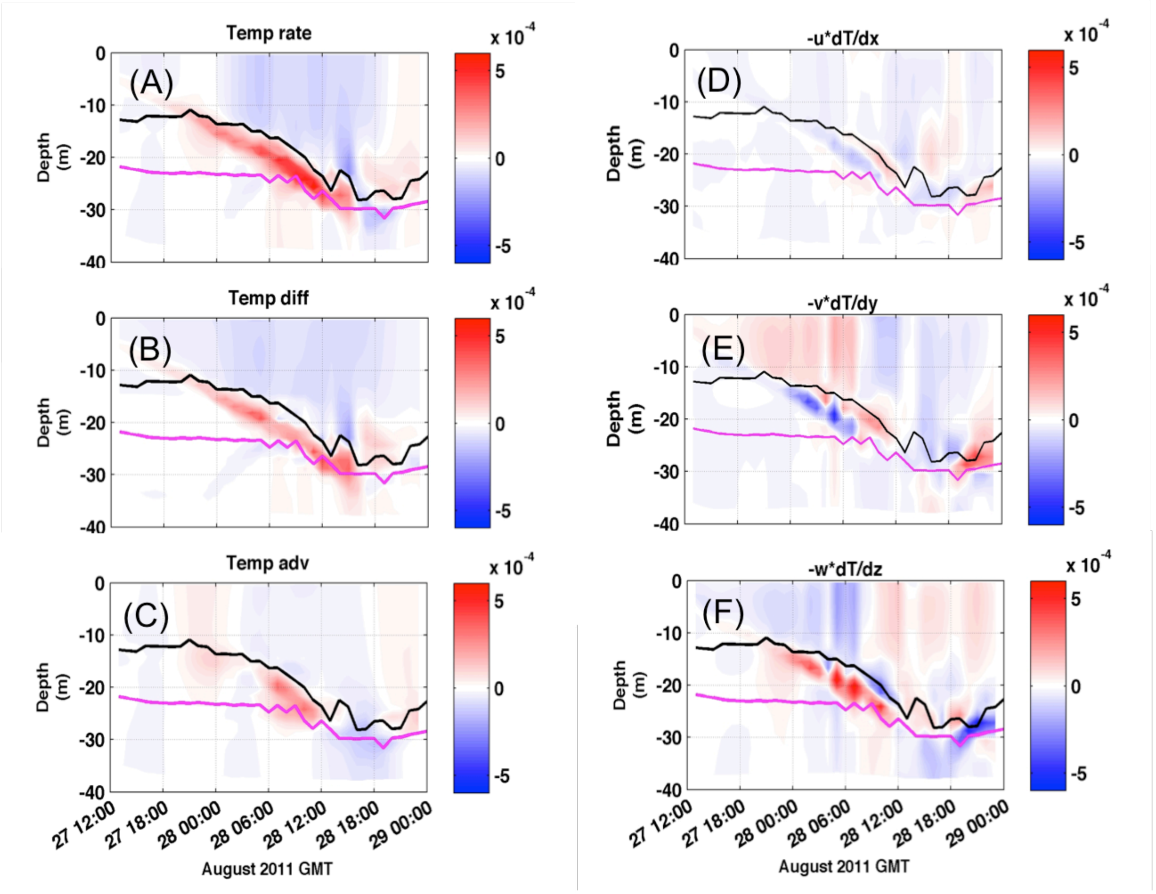

Supplementary Fig. 6 The temperature equation diagnostic terms in °C/second along the glider track from ESPreSSO ROMS model. (A) Temperature rate of change, (B) Vertical diffusion term, (C) Total advection. The total advection is separated into along-shelf advection (D, u*dT/dx); cross-shelf advection (E, v*dT/dy); and vertical advection (F, w*dT/dz). The black contour in all plots indicates the bottom of the surface mixed layer and magenta contour indicates the bottom of the thermocline. Landfall time was on August 28th 2011 at 9:35 GMT.

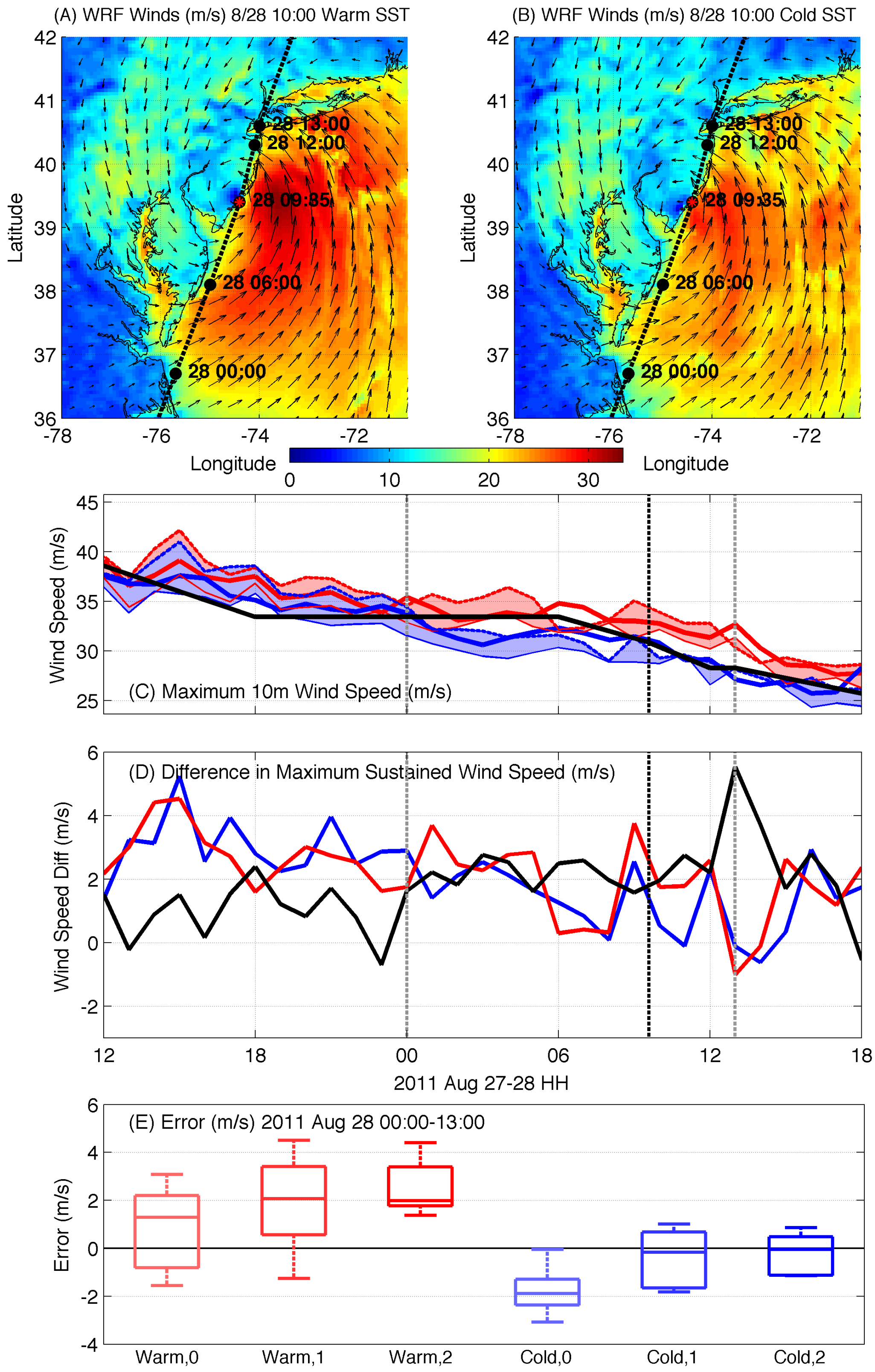

Supplementary Fig. 7 Same as Fig. 4 but for maximum sustained 10m wind speeds (m/s).

Pressure Sensitivity Table: 8/28 00-13 UTC

Group	Name	Options
Model Setup	Horizontal resolution	3km vs. 6km
	Vertical resolution	51 vs. 35 levels
	Adaptive time step	on vs. off
	B.C. (Frequency)	3 hrs vs. 6 hrs
	Digital Filter Initialization (DFI)	on vs. off
Atmospheric/ Model Physics	Microphysics	6 vs. 16
	PBL scheme	5 vs. 1
		7 vs. 1
	Cumulus parameterization	1 vs. 0
	SST skin	on vs. off
	Longwave radiation physics	1 vs. 4
		5 vs. 4
		99 vs. 4
	Shortwave radiation physics	1 vs. 4
		5 vs. 4
		99 vs. 4
	Latent heat flux <0 over water	on vs. off (warm SST)
		on vs. off (cold SST)
	Land surface physics	1 vs. 2
Advanced Hurricane WRF	Air-sea flux parameterizations	1 vs. 0 (warm SST)
		1 vs. 0 (cold SST)
Sea Surface Temperature	SST	Warm vs. Cold (isftcflx=2)
		Warm vs. Cold (isftcflx=1)
		Warm vs. Cold (isftcflx=0)

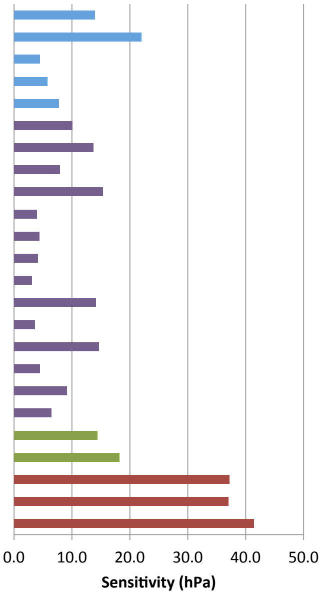

Supplementary Fig. 8 Central pressure sensitivity table. Sensitivity (hPa) is calculated by taking the cumulative hourly sum of the differences from 00 to 13 UTC on 28 August. Options column is described in Supplementary Table 1.

Wind Sensitivity Table: 8/28 00-13 UTC

Group	Name	Options
Model Setup	Horizontal resolution	3km vs. 6km
	Vertical resolution	51 vs. 35 levels
	Adaptive time step	on vs. off
	B.C. (Frequency)	3 hrs vs. 6 hrs
	Digital Filter Initialization (DFI)	on vs. off
Atmospheric/ Model Physics	Microphysics	6 vs. 16
	PBL scheme	5 vs. 1
		7 vs. 1
	Cumulus parameterization	1 vs. 0
	SST skin	on vs. off
	Longwave radiation physics	1 vs. 4
		5 vs. 4
		99 vs. 4
	Shortwave radiation physics	1 vs. 4
		5 vs. 4
		99 vs. 4
	Latent heat flux <0 over water	on vs. off (warm SST)
		on vs. off (cold SST)
	Land surface physics	1 vs. 2
Advanced Hurricane WRF	Air-sea flux parameterizations	1 vs. 0 (warm SST)
		1 vs. 0 (cold SST)
Sea Surface Temperature	SST	Warm vs. Cold (isftcflx=2)
		Warm vs. Cold (isftcflx=1)
		Warm vs. Cold (isftcflx=0)

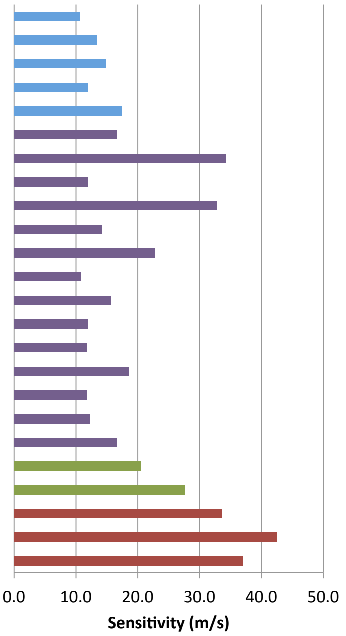

Supplementary Fig. 9 Same as Supplementary Fig. 8 but for maximum sustained 10m wind speeds (m s^{-1}). Options column is described in Supplementary Table 1.

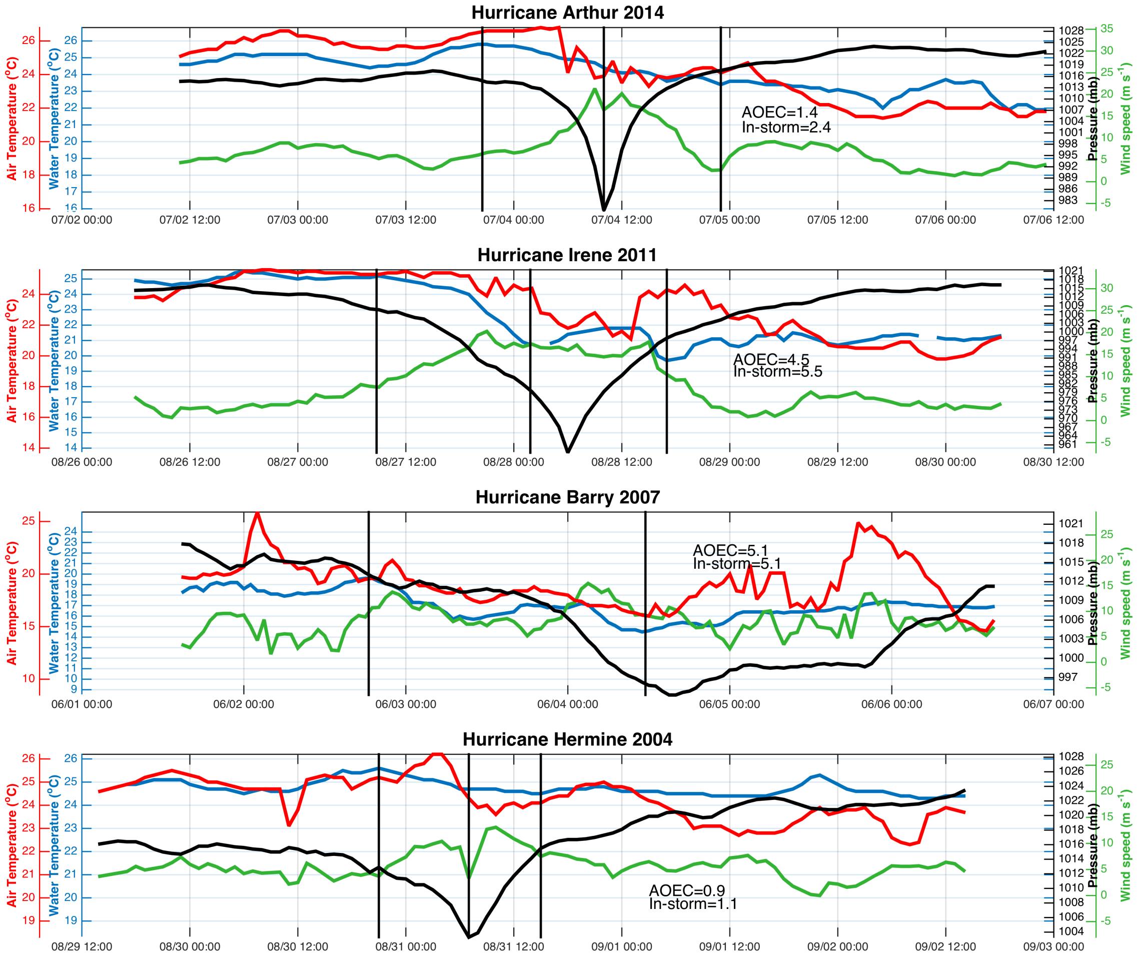

Supplementary Fig. 10 Time-series plots of air temperature (red), water temperature (blue), pressure (black), and wind speed (green). The first vertical black line indicates the maximum water temperature before wind speed increased above 5 m s^{-1}, the second represents the minimum temperature ahead-of or at the minimum pressure, and the third vertical black line is the minimum temperature before wind speeds drop below 5 m s^{-1}. Values from these plots are used to determine ahead-of-eye-center (AOEC) and in-storm cooling from NDBC buoys used to create Table 1 for Hurricanes Arthur (2014), Irene, Barry, and Hermine.

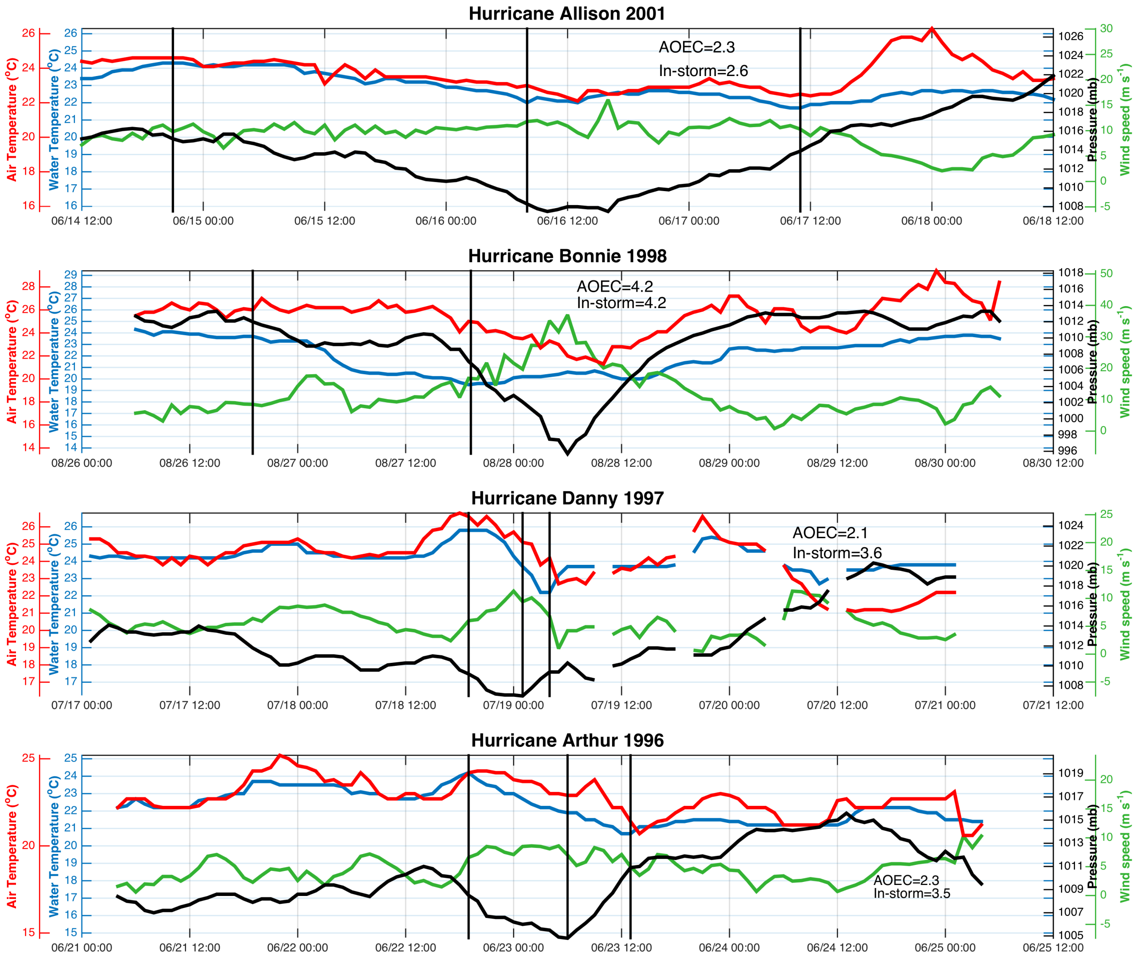

Supplementary Fig. 11 Time-series plots of air temperature (red), water temperature (blue), pressure (black), and wind speed (green). The first vertical black line indicates the maximum water temperature before wind speed increased above 5 m s^{-1}, the second represents the minimum temperature ahead-of or at the minimum pressure, and the third vertical black line is the minimum temperature before wind speeds drop below 5 m s^{-1}. Values from these plots are used to determine ahead-of-eye-center (AOEC) and in-storm cooling from NDBC buoys used to create Table 1 for Hurricanes Allison, Bonnie, Danny, and Arthur (1996).

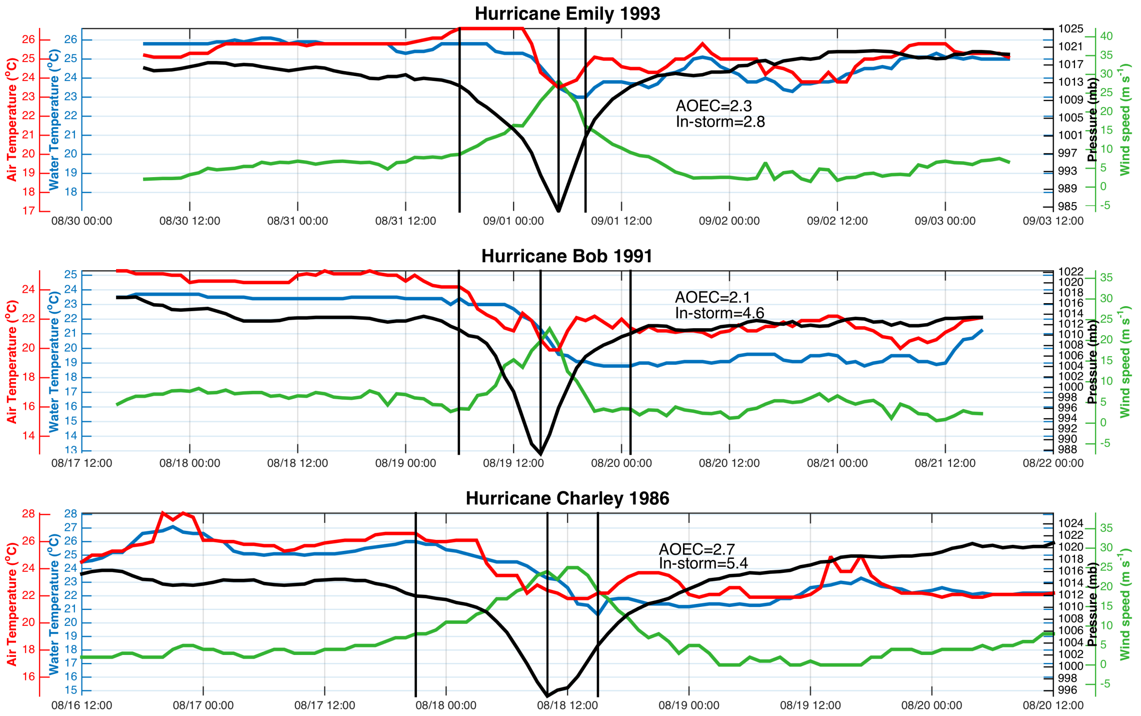

Supplementary Fig. 12 Time-series plots of air temperature (red), water temperature (blue), pressure (black), and wind speed (green). The first vertical black line indicates the maximum water temperature before wind speed increased above 5 m s^{-1}, the second represents the minimum temperature ahead-of or at the minimum pressure, and the third vertical black line is the minimum temperature before wind speeds drop below 5 m s^{-1}. Values from these plots are used to determine ahead-of-eye-center (AOEC) and in-storm cooling from NDBC buoys used to create Table 1 for Hurricanes Emily, Bob, and Charley.

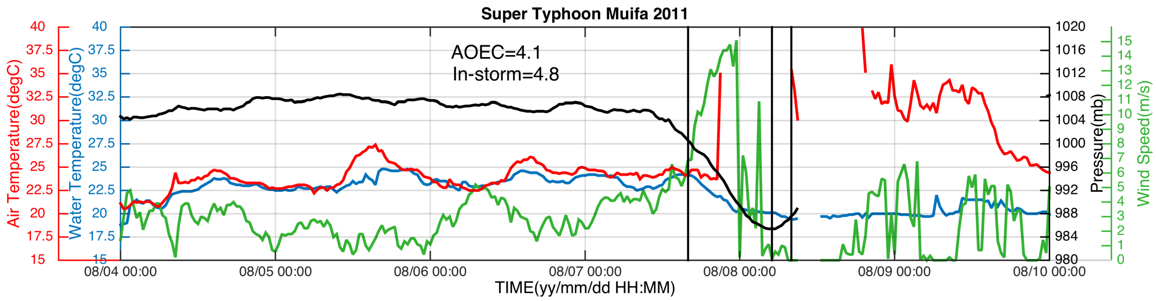

Supplementary Fig. 13 Time-series plot of air temperature (red), water temperature (blue), pressure (black), and wind speed (green). The first vertical black line indicates the maximum water temperature before wind speed increased above 5 m s^{-1}, the second represents the minimum temperature ahead-of or at the minimum pressure, and the third vertical black line is the minimum temperature before wind speeds drop below 5 m s^{-1}. This buoy was positioned in the Yellow Sea at 37.045 N 122.66 E for Super Typhoon Muifa. Values from this plot are used to determine ahead-of-eye-center (AOEC) and in-storm cooling used to create Table 1 for Super Typhoon Muifa.

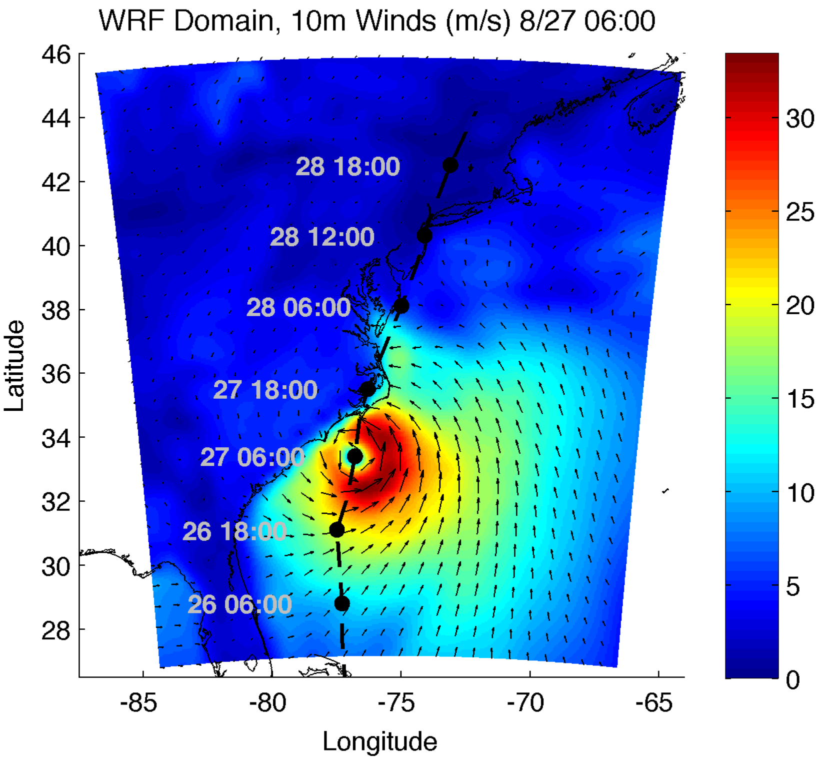

Supplementary Fig. 14 WRF 10 m winds (m s^{-1}) at model initialization time (27 August 0600 UTC) to depict model domain. NHC best track shown in black.

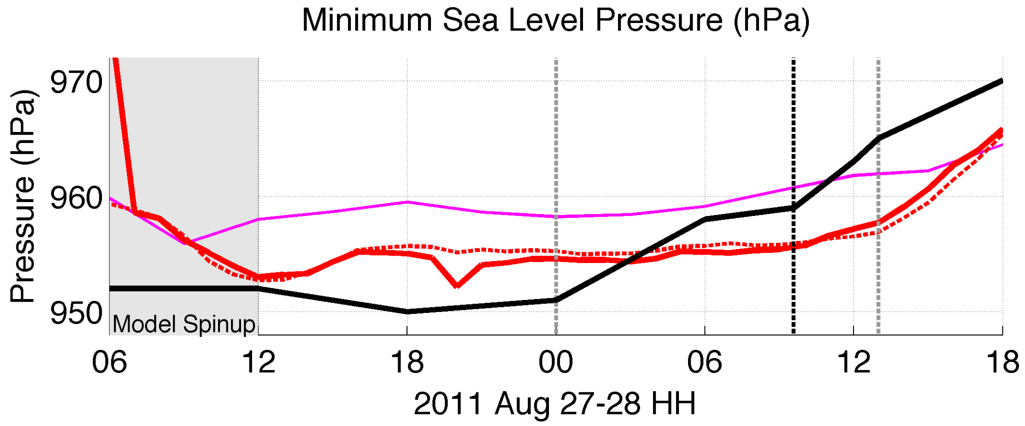

Supplementary Fig. 15 Minimum SLP for NHC best track (black), GFS 0.5° (magenta), and WRF's DFI off (red solid) and on (red dashed) with air-sea flux parameterization option isftcflx=2 for the warm SST. Vertical black dashed lines indicate eye enters MAB, makes landfall and leaves MAB. Gray shaded region indicates first 6 hours used as model spin-up period.

**Model Configuration**	**Options**
1. Horizontal resolution (*dx*)	3 km vs. 6 km
2. Vertical resolution (*e_vert, eta_levels*)	51 vs. 35 vertical levels
3. Adaptive time step (*use_adaptive_time_step*)	on vs. off
4. Boundary conditions (update frequency, *interval_seconds*)	3 vs. 6 hours
5. Digital Filter Initialization (DFI, *dfi_opt*)	on vs. off
**Atmospheric/Model Physics**	
6. Microphysics (*mp_physics*)	6 (WRF Single-Moment 6-class) vs. 16 (WRF Double-Moment 6-class)
7-8. Planetary boundary layer scheme (*bl_pbl_physics*)	5 (Mellor-Yamada Nakanishi and Niino Level 2.5) vs. 7 (ACM2) vs. 1 (Yonsei University)
9. Cumulus parameterization (*cu_physics*)	1 (Kain-Fritsch, *cudt*=0, *cugd_avedx*=1) vs. 0 (off)
10. SST skin (*sst_skin*)	on vs. off
11-13. Longwave radiation (*ra_lw_physics*)	1 (RRTM) vs. 5 (New Goddard) vs. 99 (GFDL) vs. 4 (RRTMG)
14-16. Shortwave radiation (*ra_sw_physics*)	1 (Dudhia) vs. 5 (New Goddard) vs. 99 (GFDL) vs. 4 (RRTMG)
17-18. Latent heat flux <0 over water (in *module_sf_sfclay)*	on vs. off (warm SST)<br>on vs. off (cold SST)
19. Land surface physics (*sf_surface_physics*)	1 (5-layer thermal diffusion) vs. 2 (Noah)
**Advanced Hurricane WRF (AHW) Options**	
20-21. Air-sea flux parameterizations (*isftcflx*)	1 vs. 0 (warm SST)<br>1 vs. 0 (cold SST)
**Sea Surface Temperature**	
22-24. SST	cold vs. warm (isftcflx=2)<br>cold vs. warm (isftcflx=1)<br>cold vs. warm (isftcflx=0)

Supplementary Table 1 WRF model configurations and setups for different sensitivities shown in Supplementary Figs. 8-9.
